# The Role of Cytokines in Epithelial–Mesenchymal Transition in Gynaecological Cancers: A Systematic Review

**DOI:** 10.3390/cells12030416

**Published:** 2023-01-26

**Authors:** Irene Ray, Agnieszka Michael, Lisiane B. Meira, Patricia E. Ellis

**Affiliations:** 1 Faculty of Health and Medical Sciences, School of Biosciences and Medicine, University of Surrey, Leggett Building, Daphne Jackson Road, Guildford GU2 7WG, UK; 2 Royal Surrey NHS Foundation Trust, Egerton Road, Guildford GU2 7XX, UK

**Keywords:** epithelial–mesenchymal transition, EMT, cytokines, gynaecological cancer, ovarian cancer, cervical cancer, endometrial cancer, development and progression of cancer, systematic review

## Abstract

Chronic inflammation has been closely linked to the development and progression of various cancers. The epithelial–mesenchymal transition (EMT) is a process involving the acquisition of mesenchymal features by carcinoma cells and is an important link between inflammation and cancer development. Inflammatory mediators in the tumour micro-environment, such as cytokines and chemokines, can promote EMT changes in cancer cells. The aim of this systematic review is to analyse the effect of cytokines on EMT in gynaecological cancers and discuss their possible therapeutic implications. A search of the databases CINAHL, Cochrane, Embase, Medline, PubMed, TRIP, and Web of Science was performed using the keywords: “cytokines” AND “epithelial mesenchymal transition OR transformation” AND “gynaecological cancer”. Seventy-one articles reported that various cytokines, such as TGF-β, TNF-α, IL-6, etc., promoted EMT changes in ovarian, cervical, and endometrial cancers. The EMT changes included from epithelial to mesenchymal morphological change, downregulation of the epithelial markers E-cadherin/β-catenin, upregulation of the mesenchymal markers N-cadherin/vimentin/fibronectin, and upregulation of the EMT-transformation factors (EMT-TF) *SNAI1/SNAI2/TWIST*/*ZEB*. Cytokine-induced EMT can lead to gynaecological cancer development and metastasis and hence novel therapies targeting the cytokines or their EMT signalling pathways could possibly prevent cancer progression, reduce cancer recurrence, and prevent drug-resistance.

## 1. Introduction

The epithelial–mesenchymal transition (EMT) is a process implicated in cancer progression and metastasis, whereby epithelial cancer cells lose cellular polarity and cell-to-cell adhesions and gain metastatic and invasive properties [1]. Historically, EMT was first described in the embryonic development process, also called classical EMT [2]. However, EMT was later found to be associated with various physiological processes such as wound healing, tissue regeneration, organ fibrosis, and cancer development [2]. Inflammation is another physiological process known to promote cancer development by various molecular mechanisms, EMT being one of them [3]. EMT is said to be a pivotal point between inflammation and cancer development [4]. Inflammatory mediators such as cytokines and other soluble factors, oxidative stress, or hypoxia can promote the acquisition of EMT-like features in cancer cells. In addition, cancer cells can stimulate the secretion of cytokines and pro-inflammatory molecules that foster an inflammatory tumour micro-environment (TME) creating a self-propagating habitat for cancer cell growth [3]. The TME comprises several cell types, such as tumour cells and stroma, inflammatory and immune cells, extracellular matrix components (ECM), cancer-associated fibroblasts (CAFs), tumour-associated macrophages (TAMs), endothelial cells, epithelial cells, and mesenchymal stem cells, which are involved in the upregulation of various cytokines reported to be involved in EMT [5]. However, many of the molecular mechanisms of cytokine-EMT signalling and cancer propagation are still unknown. A better understanding of the molecular pathways of cytokine-EMT signalling in cancer may help in developing targeted therapy as an additional modality complementing surgery and chemotherapy.

### 1.1. Epithelial-Mesenchymal Transition and Cancer

EMT in cancer development is characterised by the loss of cell–cell adhesion structures and a change of cell polarity. The cells also acquire a spindle shape. EMT is also characterised by the downregulation of cell–cell junction proteins, reversal of intermediate filaments from keratin to vimentin, and increased cell invasion and motility [6]. The process of EMT includes the downregulation of epithelial marker epithelial-cadherin (E-cadherin) and the relocation of β-catenin from the cell membrane to the nucleus. This results in the induction of EMT and the activation of various downstream EMT master regulators [7]. The mesenchymal markers involved in EMT are usually upregulated and they include vimentin, neural cadherin (N-cadherin), and fibronectin. Many cancer cells can initiate a process called the ‘cadherin switch’, whereby there is a reduction in E-cadherin expression and an induction of N-cadherin expression; this represents the initiation point of EMT [8].

EMT confers epithelial cancer cells with malignant potential by various mechanisms such as increasing cell motility, invasion, and metastasis, resistance to apoptosis, and acquisition of stem cell properties, all of which can promote cancer progression and may also contribute to intrinsic or acquired drug resistance [9]. Alongside EMT, ‘cancer cell plasticity’ is another closely related terminology whereby cancer cells need to constantly adapt to frequently changing and often aggressive host environmental conditions to progress from primary tumour to metastasis. Cancer cell plasticity is brought about by back-and-forth transitions from differentiated to undifferentiated cellular morphology or EMT-associated changes. [10]

The transcriptional repression of E-cadherin and induction of N-cadherin can be achieved by several EMT-activating transcription factors (EMT-TFs), such as zinc finger Snail homologs (*SNAI1*/Snail, *SNAI2*/Slug, and *SNAI3*/Smuc) and helix–loop–helix factors (*TWIST1*, *TWIST2*, *ZEB1*, *ZEB2*, etc.) [7]. EMT-TFs are crucial mediators of cellular plasticity, which is essential for cancer progression and metastasis [10]. The Snail family TFs, *SNAI1* and *SNAI2*, downregulate the expression of various genes related to EMT, most prominently E-cadherin [11]. Apart from promoting EMT, Snail members also promote cell survival, block cell cycle, inhibit apoptosis, and help in acquiring stem cell features. The Twist family TFs, *TWIST1* and *TWIST2*, have important physiological roles during embryonic morphogenesis, wound healing, and tissue fibrosis [12]. Their expressions are upregulated during cancer development and can induce EMT [12]. Increased expression of *TWIST1* is directly associated with tumour invasion and metastasis and mediates the loss of E-cadherin and increases the expression of mesenchymal markers fibronectin, N-cadherin, and vimentin. *ZEB1* and *ZEB2* actively downregulate epithelial cell markers and upregulate mesenchymal markers, hence promoting EMT [13].

However, during cancer development and progression, the transcriptional factors and pathways mentioned above may not all be fully activated leading to partial EMT, where epithelial cells may lose some of the epithelial characteristics or develop both epithelial and mesenchymal characteristics [10]. Moreover, EMT-TFs are pleiotropic and may have other functions besides promoting cancer progression and metastasis. Additionally, not all EMT markers/EMT-TFs are activated in all cancers and different TFs may be involved in EMT depending on the organ of origin of cancer. Differential expressions of EMT markers and EMT-TF involvement were reported across the literature in various cancers such as prostate, lung, liver, pancreatic, and breast cancers [14,15,16,17,18,19,20,21,22]. Metastatic prostate cancer cells were associated with the decreased expression of E-cadherin [14] and were found to be an independent predictor for tumour relapse [15]. *TWIST* was correlated with metastasis in hepatocellular carcinoma and its expression was negatively correlated with E-cadherin expression [16]. E-cadherin and β-catenin were found to be reduced in lung cancer and a reduction in both led to a significantly unfavourable prognosis [17] and was found to be associated with increased expression of EMT-TFs, *SNAI2*, and *ZEB1* [18]. In addition, vimentin and *SNAI1* have also been associated with the malignant phenotype of non-small cell lung cancer (NSCLC). The nuclear translocation of β-catenin was reported in a study on NSCLC [19]. In a pancreatic cancer model study by Zheng et al., *SNAI1*- or *TWIST*-induced EMT was not found to be essential for the invasion and metastasis of pancreatic cancer, but they suggested that potentially targeting EMT may enhance the efficacy of chemotherapy and immunotherapy [20]. A further study using another pancreatic cancer model demonstrated that *ZEB1* promoted the invasion and metastasis of cancer cells and the depletion of *ZEB1* suppressed EMT-related cancer promoting changes [21]. In a study on breast cancer, *SNAI1* was found to be an EMT-inducing factor as it downregulated E-cadherin, upregulated vimentin, and induced classical morphologic changes of EMT [22]. In addition to a varied expression in different cancers, the transcription factors could have antagonistic functions as well. A study on EMT in malignant melanoma reported that *SNAI2* and *ZEB2* transcription factors are expressed in normal melanocytes and behave as tumour-suppressor proteins, whereas *TWIST1* and *ZEB1* favour neoplastic transformation in melanocytes [23]. The data on the effects of EMT reversal on cancer progression/spread and drug resistance are not yet substantial, so we need robust studies to assess if reversing EMT can inhibit invasion and metastasis in cancers and counteract drug resistance.

### 1.2. Cytokines and EMT in Cancer Progression

Various cytokines and chemokines produced by tumour cells, CAFs, or tumour-associated immune cells in the TME can stimulate the EMT process and thereby promote cancer cell metastasis. In addition, EMT-TFs have been shown to upregulate the expression of pro-inflammatory and immunosuppressive cytokines such as TGF-β and IL-10 in cancer cells, thereby triggering tumour promoting effects on the TME.

A variety of metastasis-related chemokines (including CCL5, SDF-1, CCL2, and CCL7) and cytokines (such as IL-1, IL-6, IL-8, and TNF-α) can be released from cancer stem-like cells (CSLCs) [24]. IL-1β was reported to promote EMT in colon cancer through *ZEB1* activation [24]. Another study reported that the IL-8 secreted by tumour cells undergoing EMT could then potentiate tumour progression by inducing adjacent epithelial tumour cells into EMT [25]. Onoue et al. performed a study on the effect of chemokines on EMT and demonstrated that a stromal cell-derived factor-1 (SDF-1)/CXCR4 system can facilitate lymph node metastasis in oral squamous cell carcinoma (SCC) [26]: SDF-1/CXCR4, via the activation of Phosphoinositide 3-kinase-protein kinase B (PI3K-Akt/PKB) pathway, was associated with the loss of epithelial cell morphology alongside the downregulation of epithelial markers, cytokeratin, E-cadherin, and beta-catenin and the upregulation of mesenchymal markers, vimentin, and Snail [26]. TGF-β is one of the main inducers of EMT in several biological systems [27]. Recent studies have identified two main classes of signalling pathways that are responsible for the mediation of EMT by TGFβ1: the canonical Smad signalling pathway and various non-canonical Smad-independent pathways, including the extracellular signal-regulated kinase 1/2 (ERK1/2), PI3K, c-Jun N-terminal kinase (JNK), and P38-mitogen-activated protein kinase (MAPK) pathways [28]. TGFβ1 induces *SNAI1* expression in several cell types including hepatocytes, palate, and mesothelial cells. In a study on human NSCLC, IL-27 was found to induce mesenchymal morphological changes in IL-27-treated NSCLC cells, reduce epithelial markers (E-cadherin and γ-catenin) and EMT-TF Snail, and reciprocally increase mesenchymal marker vimentin predominantly through the Janus kinase-signal transducer and activator of the transcription1 (JAK/STAT1) pathway. The STAT1 pathway was implicated in the EMT changes induced by IL-27 as STAT1 inhibition reversed the EMT effects of IL-27 on IL-27-treated cells [29]. IL-6 was found to downregulate E-cadherin expression in breast cancer [30,31] and activate EMT-TF *TWIST* [31].

In multiple carcinomas, EMT has been demonstrated to be regulated by cytokine secretion and is possibly involved in cancer progression and metastasis including gynaecological cancers. There is a high level of drug resistance and mortality in gynaecological cancers due to metastasis and recurrence despite advances in modern diagnostic and treatment modalities. As EMT plays a crucial role in cancer metastasis, understanding the mechanism of action of the underlying EMT factors and their signalling pathways may help in reducing morbidity, mortality, and drug resistance associated with gynaecological cancers. We conducted this systematic review to summarise the evidence so far on the effect of cytokines on EMT in gynaecological cancers.

## 2. Objectives

The aim of this systematic review is to describe the effect of cytokines on EMT in gynaecological cancers and their possible therapeutic implications. This will be achieved by reporting correlations between cytokines and markers of EMT in gynaecological cancers such as morphological changes (acquisition of spindle shape), suppression of epithelial markers (E-cadherin/catenin), upregulation of mesenchymal markers (N-cadherin/vimentin/fibronectin), and association with EMT TFs—*SNAI1*/*SNAI2*/*TWIST*/*ZEB*. The possible pathways of EMT (if mentioned in the studies) will also be documented for their potential therapeutic importance.

## 3. Materials and Methods

### 3.1. Protocol and Registration

A protocol for the review was devised and registered with PROSPERO (Registration No. CRD42022358266).

### 3.2. Eligibility Criteria

#### 3.2.1. Inclusion Criteria

(a) English language studies from January 2000 to December 2021.
(b) Studies including human subjects and cancer cell lines.
(c) Experimental study design.
(d) Exposure of interest: cytokines and chemokines only.
(e) Disease of interest: Gynaecological cancers
(f) Outcome of interest: EMT
(g) Outcome measure: Effect of cytokines on EMT such as mesenchymal morphological changes (acquisition of spindle shape), suppression of epithelial markers (E-cadherin/catenin), upregulation of mesenchymal markers (N-cadherin/vimentin/fibronectin), and association with EMT TFs—*SNAI1*/*SNAI2*/*TWIST*/*ZEB*. The possible pathways of EMT (if mentioned in the studies) will also be noted for their potential therapeutic importance.

#### 3.2.2. Exclusion Criteria

(a) Review articles on the subject/full text article not available.
(b) The cancer under study is not a gynaecological cancer.
(c) Studies involving the effect of any other markers and not cytokines on EMT in gynaecological cancers.
(d) No mention of the effect of cytokines on EMT in gynaecological cancers.
(e) Animal studies (however, studies with both human and animal study arms have been included in the systematic review without going into any description of the animal study arm).

### 3.3. Information Sources

A search of the databases CINAHL, Cochrane, Embase, Medline, PubMed, TRIP, and Web of Science were performed to identify the relevant keywords contained in the titles and abstracts. A grey literature search was also performed to search for relevant conference abstracts, book chapters, leaflets, and dissertations.

### 3.4. Search Strategy and Selection Process

The search criteria keywords were: “cytokines” AND “epithelial mesenchymal transition OR transformation “AND “gynaecological cancer” (endometrial cancer OR ovarian cancer OR cervical cancer OR vulval cancer OR vaginal cancer). The individual search strategies are included at the end (Supplementary Materials).

Articles published in the last 22 years (from January 2000 to August 2022) in English that are indexed in the above databases were identified based on their titles and abstracts. After a perusal of the titles, duplicate studies were excluded, the abstracts for the remaining articles were reviewed, and the articles not satisfying the inclusion criteria strictly were discarded. Thereafter, full texts were obtained for the outstanding articles included so far based on their abstracts. These articles were then screened for relevance and inclusion in the systematic review for data extraction and synthesis.

The eligibility of each study was checked independently by two reviewers (I.R. and P.E.E.). The lists of included studies selected by the two reviewers were then compared and any disagreement was resolved through discussion with an independent third reviewer (L.B.M).

**Figure 1:**
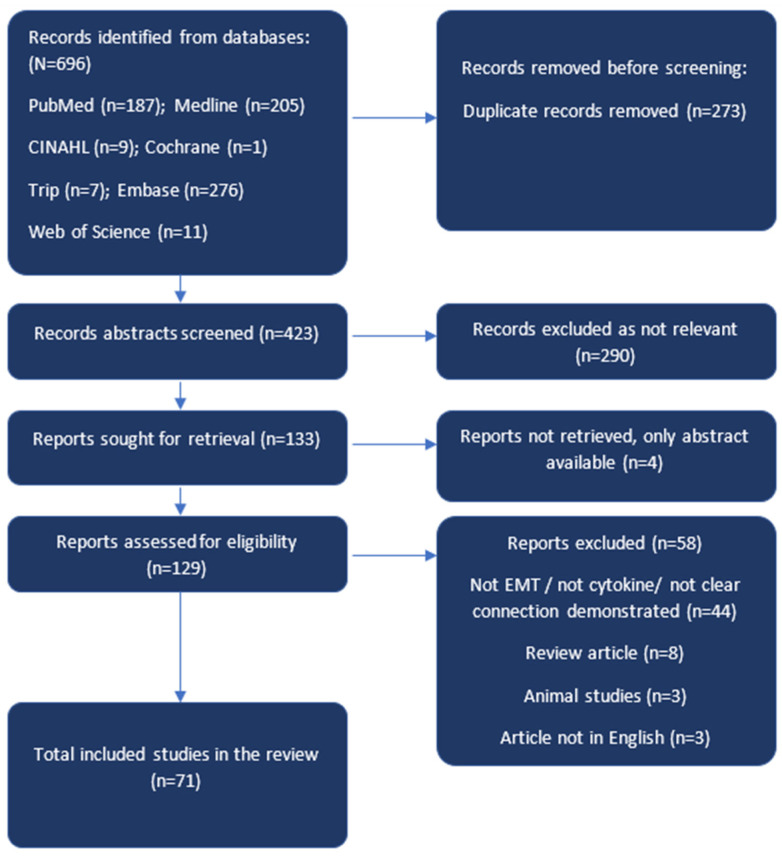
Flow diagram showing the stepwise process of screening, exclusion, inclusion, and analysis of articles as per the Preferred Reporting Items for Systematic Reviews and Meta Analyses Protocols (PRISMA) 2020 [32] guidelines.

The search for articles for the study was performed following the new 2020 PRISMA guidelines (Preferred Reporting Items for Systematic Reviews and Meta Analyses Protocols) [32] and is presented in the form of a flow diagram (Figure 1).

### 3.5. Data Extraction

The data from each study included in the review were extracted by two independent reviewers (I.R. and P.E.E.). The extracted data elements included: first author’s name, publication year, study country, sample population—human samples/cell lines, cytokines/chemokines, type of gynaecological cancer studied, laboratory assays, effect of cytokine on EMT, EMT markers acquired, the possible pathways of EMT (if mentioned), and potential therapeutic implications.

### 3.6. Risk of Bias in Individual Studies

The studies were assessed independently for their content and methodological validity by two reviewers (I.R. and P.E.) prior to inclusion in the review. Any disagreement was resolved through discussion with an independent third reviewer (L.B.M.). The studies included were assessed for their ethical conduct and sourcing of materials.

### 3.7. Synthesis of Results

The studies that satisfy the inclusion criteria were divided into broad groups based on the type of gynaecological cancer: ovarian, cervical, and endometrial. No studies were found for vaginal and vulval cancers that satisfied the inclusion criteria.

After dividing into the initial three cancer groups, the findings of the studies were extracted from each study’s results and discussion sections and grouped into clusters based on the EMT changes such as the epithelial–mesenchymal morphological change, downregulation of epithelial markers (E-cadherin/β-catenin/claudin/any other EMT-related epithelial marker), upregulation of mesenchymal markers (N-cadherin/vimentin/fibronectin/any other EMT-related mesenchymal marker), and alteration of EMT-TFs (*SNAI1*/*SNAI2*/*TWIST*/*ZEB*). After dividing the EMT changes brought about by cytokines into sub-group clusters, general conclusions were drawn for each cluster in the form of narrative synthesis.

## 4. Results

### 4.1. Search Results and Publication Characteristics

There were 696 articles published in the last 22 years (from January 2000 to August 2022) in English that were indexed in the above databases and were identified based on their titles and abstracts. Before screening for duplicate records, 273 studies were excluded; 423 studies were screened using their title and abstract and then 290 studies were excluded as they were not relevant to our review search criteria. Full texts were sought for the remaining 133 articles, among which 4 were conference abstracts only, so no full texts could be found and hence discarded. Next, 129 full text articles were screened for relevance and inclusion in the systematic review. A further 58 studies were excluded as they were not exactly relevant to our topic of interest, were animal studies, or did not clearly demonstrate a correlation between any cytokine and EMT changes in a gynaecological cancer. Finally, 71 articles were included in the review for data extraction and synthesis (flow diagram Figure 1). The characteristics of these studies are presented in Table 1 in the order of their year of publication and grouped with respect to the type of gynaecological cancer.

### 4.2. Ovarian Cancer

In this systematic review, forty-five studies on ovarian cancer have been included that satisfied the inclusion criteria and demonstrated that cytokines induce EMT changes in ovarian cancer. Various human ovarian cancer cell lines such as A2780, SKOV-3, ES-2, HEY, IGROV-1, HO-8910, and OVCAR3 or human ovarian tissues have been assessed for EMT changes. Table 2 summarises the effect of cytokines/chemokines and their mechanism of EMT in ovarian cancer. To simplify our findings, the EMT changes have been classified under the following sub-headings:

*Mesenchymal morphological changes:*

Mesenchymal morphological changes of ovarian cancer cells such as changes of cell shape from cobblestone (epithelial) to spindle-like narrow elongated shape (mesenchymal) have been implicated as a marker of EMT and has been reported to be induced in ovarian cancer by treatment with cytokines such as TGF-β [44,51,58,62,70,75], TNF-α [51], and chemokine CXCL12 and its receptor CXCR4 [52,53].

*Downregulation of epithelial markers:*

Various studies on EMT in ovarian cancer have demonstrated a downregulation of the epithelial markers E-cadherin and β-catenin induced by cytokines either in their genetic expression or protein expression or both.

Reduced expression of E-cadherin has been reported in ovarian cancer cells following TGF-β treatment [33,36,39,41,43,46,48,49,50,51,54,55,56,58,60,61,62,64,70,71,72,73,74,75,76], treatment with bone morphogenetic protein (BMP)-9, a member of the TGF-β superfamily [42], IL-6 treatment [34,47], IL-8 treatment [37,66], IL-17 treatment [38], and TNF-α treatment [51] as well as treatment with chemokine CXCL12 and its receptors CXCR4 [52], CCL5 [67], CCL19, and CXCR7 [68,69]. The epithelial marker β-catenin is also reported to be downregulated similarly in ovarian cancer cells by treatment with TGF-β [40], but β-catenin was found to be upregulated by IL-8 [37,66] and nuclear translocation of β-catenin, which is also a form of change expected with EMT as reported in IL-6 treatment [77]. In another study, Claudin, an epithelial marker, had reduced expression after TGF-β treatment of ovarian cancer cells [43,55].

*Upregulation of mesenchymal markers:*

Mesenchymal markers such as vimentin/fibronectin/N-cadherin have been reported to be over-expressed either at the genetic level or at the protein level on treating ovarian cancer cells or cell lines in various studies.

Vimentin has been reported to be upregulated [33,35,36,40,41,43,46,49,55,58,61,62,64,70,71,73] or partially upregulated by treatment with TGF-β [39], IL-6 [34,47,77], IL-8 [37], or IL-17 treatment [38] or when treated by chemokines such as CXCL12/CXCR4 [52], CCL5 [67], or CCL19/CXCR7 [68]. Fibronectin has been reported to be over-expressed as a part of EMT induced by TGF-β [39,41,56,57]. N-cadherin, another mesenchymal factor has been reported to be over expressed as a part of EMT by treating with TGF-β [35,36,40,41,43,46,48,49,50,51,54,60,61,64,70,74,75], BMP [42], IL-6 [34,47,77], TNF-α [51], or chemokines, such as CXCL12/CXCR4 [52] or CCL19/CXCR7 [68,69].

*EMT-TF activation/suppression:*

EMT TFs such as *SNAI1*/2, *ZEB*, and *TWIST* have been reported to be upregulated in ovarian cancer cell lines when treated with various cytokines as discussed below.

*SNAI2*/Slug [35,40,43,54,58,62,73,74,76], and *SNAI1*/Snail [35,41,43,54,58,61,62,64,72,74,76] have been reported to be upregulated by treatment with TGF-β and BMP [42] as well as by treating ovarian cancer cells with IL-17 [38], chemokine CCL5 [67], and CCL19/CXCR7 [68,69]. *TWIST* has been reported to be upregulated by treatment with IL-6 [34], TGF-β [48,51,54,62,74], BMP [42], and lL-17 [38]. *ZEB1* has been upregulated by TGF-β [43,44,54,74] and ZEB2 by TGF-β [43,56,62] treatment of ovarian cancer cells.

*Pathways and interactions:*

TGF-β has been reported to be involved in EMT in ovarian cancer by various pathways in the studies included in Table 1. The signalling pathways reported are WNT/β-catenin pathway [55] and SMAD2/3 signalling activation [40,41,75]. Left–right determination factor (LEFTY), a member of the TGF-β superfamily, was reported to be involved in the TGF-β/Smad/*SNAI1* signalling for EMT induction in ovarian clear cell carcinoma cells [46]. Xu et al. [76] demonstrated that TGF-β and Epidermal growth factor (EGF) signalling pathways synergistically induce EMT and render epithelial ovarian cancer cells a more invasive phenotype. Matrix metalloproteinases (MMP2/9) are involved with cell motility and are closely related to EMT and were reported to be upregulated by TGF-β in various studies [39,40,41,56,76]. A study by Ren et al. [36] demonstrated that TGF-β treatment decreased miRNA-200 expression. MicroRNAs (miRNAs) are small non-coding single-stranded RNAs that control gene expression by targeting mRNA translation. MiRNA-200 has been reported to have a suppressive effect on EMT by inhibiting transcriptional repressors ZEB1 and ZEB2. The authors reported that miRNA-200 inhibited EMT by downregulating the sex-determining region Y-box 4 (SOX4), which is an upstream factor for EMT. They also demonstrated that TGF-β treatment induced EMT by decreasing miRNA-200 expression. The TGF-β/ZEB/miR-200 signalling pathway, an autocrine regulatory network, has been reported by Gregory et al. [104] to control the plasticity between the epithelial and mesenchymal states of the cells. TGF-β signalling activated ZEB1/2, which in turn induced EMT by repressing epithelial genes. Furthermore, miRNA-200 was noted to suppress ZEB1/2 and promote epithelial differentiation. ZEB1/2 knockdown enhances miRNA-200 expression and TGF-β signalling is a target of miRNA-200. However, exogenous TGF-β administration can inhibit miRNA-200. Hence, there is an interconnection between TGF-β, miRNA-200, and *ZEB* that is essential in regulating the epithelial and mesenchymal reversible states [105].

Similar to TGF-β, IL-6 has been reported to be involved in EMT by various pathways. Wang et al. [47], Ma et.al. [48], and Colomiere et al. [77] reported that IL-6 mediated the EMT in OVCAR3 cells via the JAK2/STAT3 pathway. IL-6 secretion was reported to be induced by CAFs in the TME [47] and the IL-6/IL-6R/STAT3 signalling was reported to be induced by CD146 by Ma et.al. [48] and by the epidermal growth factor (EGF) by Colomiere et.al. [77].

The IL-8 and IL-8 receptors CXCR1 and CXCR2 were reported by Wen et al. [37] and Yen et al. [66] to induce EMT changes in ovarian cancer cells potentially by the Wnt/β-catenin pathway, similarly to TGF-β.

IL-17 treatment possibly induced EMT in ovarian cancer cell lines via the expression of metastasis-associated genes-1 (MTA1) and targeting the IL-17/MTA-1 axis could be used as a treatment for ovarian cancer [38].

GDF8, which belongs to the TGF-β superfamily, was reported by Zhou et al. [63] to promote EMT in ovarian cancer cells via Activin such as the kinase 4 and 5 (ALK4 and 5) pathways.

Chemokines have also been reported to activate various EMT signalling pathways. The NF-κB signalling pathway has been implicated in CCL5-induced EMT changes in ovarian non-cancer stem-cell-like cells [67] and the AKT/ERK pathway was reported to be activated by CXCR7 and its ligand CCL19 in studies by Yu et al. [68] and Cheng et al. [69].

Therapeutic possibilities:

A number of molecules or factors have been implicated in the studies included in this systematic review to either potentiate or abrogate cytokine-mediated EMT signalling and hence targeting them could open new therapeutic avenues.

In a study by Cheng et al. [74], TGF-β-induced EMT changes were demonstrated in serous borderline ovarian tumour (SBOT) cells, indicating that TGF-β-induced EMT is possibly involved in the progression from non-invasive SBOT to invasive low grade ovarian cancer (LGC) and that targeting the TGF-β signalling pathway could prevent the progression from borderline ovarian tumour to ovarian cancer. However, Sicard et al. [35] reported that TGF-β-induced mesenchymal changes were limited to the chemo-sensitive ovarian cancer cells and Ameri et al. [39] observed that TGF-β-mediated EMT was more prominent in epithelial-like ovarian cancer cell lines than invasive ovarian cancer cell lines. Therefore, strategic targeting of TGF-β signalling in non-invasive or chemo-sensitive ovarian cancers may have an increased therapeutic benefit. TGF-β has been demonstrated to induce EMT changes in ovarian cancer cells by inducing DNA methyltransferases (DNMT) that are involved in DNA methylation [71]. DNA methylation has been implicated in suppressing various EMT genes. Treatment with DNMT inhibitor (SGI-110) prevented TGF-β induced EMT changes. Hence, targeting DNMT may reverse the EMT changes or EMT gene suppressions caused by DNA methylation in ovarian cancer [71].

Additionally, TGF-β signalling has been abrogated by the use of various inhibitor molecules that could be used as a treatment for ovarian cancer by halting TGF-β-mediated EMT. These include Sorafenib, a pan-protein kinase inhibitor [33]; LY364947, a TGF-β-receptor kinase I inhibitor [39]; A83–01, a TGF-β type I receptor (TβR I) inhibitor [41]; DAPT, a γ-secretase inhibitor [54]; DKK1, a WNT signalling inhibitor [55]; SD208, a TGF-β receptor I serine threonine kinase inhibitor [75]; and BEZ235, a dual PI3K/mTOR inhibitor (currently in phase 1/2 clinical trials) [72].

Similarly, other substances have also been demonstrated by various authors to downregulate TGF-β-mediated EMT and hence may have therapeutic benefits, including Chrysin, a bio-active flavonoid [51], TET3, a ten–eleven translocator involved in DNA demethylation [61], micro-RNAs such as miRNA-200a [36] and miRNA-30d [64]; and Krüppel-like factor 4 (KLF4), which is a zinc-finger-containing transcription factor [73].

Some molecules have been shown to potentiate TGF-β signalling and, hence, targeting these molecules directly or alongside TGF-β could open new therapeutic avenues for ovarian cancer treatment. Examples of such substances in the studies included in this systematic review are: FXYD domain-containing ion transport regulator 5 (FXYD5), a cancer-associated protein [40]; ST3GAL1, a sialyl transferase [49]; and ATP-binding cassette transporter A7 (ABCA7), which is involved in the transport of various inflammatory mediators and lipids [50]. Some other molecules or genes that similarly promoted TGF-β signalling and hence could be potential therapeutic targets are—Vasohibin-2 (VASH2), an endothelium-derived angiogenesis inhibitor [56]; heat shock transcription factor 1 (HSF1), a transcription factor promoting the heat-shock response that encourages recovery from cellular damage [57]; 1α,25(OH)2D3 (Vitamin D3) [58]; Hematopoietic Pre-B-cell leukaemia transcription factor (PBX)-interacting protein (HPIP/PBXIP1), a nucleo-cytoplasmic shuttling protein [60]; Id-1, an inhibitor of differentiation or DNA-binding protein (belonging to the helix–loop–helix family of transcription factors) [70]; and, Tissue transglutaminase (TG2), a multifunctional enzyme that catalyses the crosslinking of proteins [75].

BMP9, a member of the TGF-β superfamily, has been demonstrated by Wang et al. [42] to promote EMT changes in a dose-dependent manner; the authors have also commented that BMP9-induced EMT may be partially responsible for BMP9-induced Cisplatin chemoresistance in ovarian cancer. This raises the possibility that BMP9 could be a novel therapeutic target to improve cisplatin sensitivity in chemo-resistant patients [42].

In the case of IL-6-induced EMT signalling in ovarian cancer, receptor interacting protein serine/threonine kinase 4 (RIPK4) has been reported to be of significance. RIPK4 is a key member of the group of Receptor Interacting Proteins (RIPs) and is aberrantly expressed in multiple cancer types [34]. Silencing RIPK4 significantly downregulated IL-6-mediated EMT changes [34] and, hence, dual targeting of RIPK4/IL6 could have a therapeutic advantage. Wang et al. reported that IL-6-induced EMT can enhance paclitaxel resistance in ovarian cancer cells [47] and, hence, it is possible that reversing the IL-6-mediated EMT may reverse drug resistance. However, substantial studies are needed to verify this possibility.

**Table 2:** EMT changes associated with cytokines/chemokines in ovarian cancer.

Cancer	Cytokine	Mechanism of EMT
Ovarian	TGF-β	Epithelial downregulation: E-cadherin [33,36,39,41,43,48,49,50,51,54,55,56,58,60,61,62,63,70,71,72,73,74,75,76], β-catenin [40], and claudin [43,55]. Mesenchymal upregulation: Vimentin [33,35,36,39,41,43,49,55,58,61,62,70,73], fibronectin [39,41,57], and N-cadherin [35,36,40,41,43,46,48,49,50,51,54,60,61,64,70,74,75]. EMT-TF activation/suppression: *SNAI1*/Snail [35,41,43,54,58,61,62,63,72,74,76], *SNAI2*/Slug [35,40,43,54,56,58,62,63,73,74,76], *TWIST* [48,51,54,74], *ZEB1* [43,44,62,74,75], and *ZEB2* [43,56,62]. Mesenchymal morphological changes: [62,70,71,75].
	BMP	Epithelial downregulation: E-cadherin [42]. Mesenchymal upregulation: N-cadherin [42]. EMT-TF activation: *SNAI1*, *SNAI2*, and *TWIST* [42].
	IL-6	Epithelial downregulation: E-cadherin [34], β-catenin nuclear translocation [77]. Mesenchymal upregulation: Vimentin [34,46,47]. EMT-TF activation/suppression: *TWIST* [34].
	IL-8	Epithelial downregulation: E-cadherin [37,66] and β-catenin upregulation [37,66]. Mesenchymal upregulation: Vimentin [37].
	TNF-α	Epithelial downregulation: E-cadherin [51]. Mesenchymal upregulation: N-cadherin [51]. EMT TF activation: *TWIST* [51].
	CXCL12/ CXCR4	Epithelial downregulation: E-cadherin [52]. Mesenchymal upregulation: Vimentin [52] and N-cadherin [52]. Mesenchymal morphological changes: [52,53].
	CCL5	Epithelial downregulation: E-cadherin [67]. Mesenchymal upregulation: Vimentin [67]. EMT-TF activation/suppression: *SNAI1* [67].
	CCL19/CXCR7	Epithelial downregulation: E-cadherin [68,69]. Mesenchymal upregulation: Vimentin [68] and N-cadherin [68,69]. EMT-TF activation/suppression: *SNAI1* [68,69].

### 4.3. Cervical Cancer

Eighteen studies on cervical cancer have been included in this systematic review that satisfied the inclusion criteria. Various EMT changes have been demonstrated by the treatment of cervical cancer cell lines such as C33a, Hce1, HeLa, CaSki, SiHa, etc., or human cervical cancer tissue with different cytokines/chemokines by immunoblot, immunofluorescence, or qRT-PCR. Table 3 summarises the EMT changes associated with cytokines/chemokines in cervical cancer. The EMT changes in cervical cancer have been classified under sub-headings, as follows:

*Mesenchymal morphological changes:*

Morphological change of cervical cancer cells—change of cell shape from cobblestone (epithelial) to spindle-like narrow elongated shape (mesenchymal)—has been implicated as a marker of EMT and reported to be induced in cervical cancer cells by treatment with TGF-β [78,86,88,89,90,92,95] as well as TNF-α [86].

*Downregulation of epithelial markers:*

A reduced expression of E-cadherin was reported as a result of TGF-β treatment of cervical cancer cells [78,80,81,82,83,84,85,86,88,89,90,91,92,95], TNF-α treatment [86], IL-6 treatment [94], and treatment with Chemokine (C–C motif) ligand 20 (CCL20) [93]. β-catenin expression was reduced with TGF-β treatment [82] and another epithelial marker ZO-1 was downregulated by TGF-β treatment [84,87].

*Upregulation of mesenchymal markers:*

Vimentin was found to be upregulated by treatment of cervical cancer cells with TGF-β [80,83,85,88,89,90,92]; IL-6 [94]; and with chemokine CCL20 [93]. Fibronectin was over-expressed by TGF-β treatment [84,85,87,89,95]. N-cadherin was upregulated when cervical cancer cells were treated with TGF-β [78,81,82,86,91], TNF-α [86], and chemokine CCL20 [93].

*EMT-TF activation/suppression:*

*SNAI2*/Slug [78,80,83,87] and *SNAI1* [80,83,84,87] were reported to be upregulated by TGF-β treatment of cervical cancer cells. *TWIST* was upregulated by treatment with TGF-β [78,80,83,86] and TNF-α [86], whereas ZEB-TF was over-expressed by TGF-β treatment [78,80].

*Cytokine with anti-tumour effect:*

IL-9, a T-helper 9 cytokine, reduced the expression of N-cadherin and vimentin in cervical cancer cells and increased expression of E-cadherin. Therefore, IL-9-based therapy has the potential to prevent progression and metastasis in cervical cancer [79].

*Pathways and interactions:*

Various pathways have been implicated in cytokine-induced EMT in cervical cancer. Smad2/3 signalling was found to be involved in TGF-β-induced EMT in cervical cancer cell lines [78,80,82,87,89]. Fan et al. [106] reported that TWIST EMT-TF controlled EMT induction via TGF-β/Smad3 signalling. Dong et.al. [86] suggested that TGF-β and/or TNF-α treatment induced EMT changes in cervical cancer cells via NF-κB axis. Cheng et al. reported that TGF-β1 induced EMT in tumour cells through mammalian targeting of the Rapamycin/p70s6k/Pyruvate Kinase M2 (mTOR/p70s6k/PKM2) pathway [88,90].

Miao et al. demonstrated that STAT3 is involved in IL-6-induced EMT changes in HeLa and C33A human cervical cancer cells and STAT3 silencing led to a reversal of IL-6-induced EMT changes [94].

Zhang et al. reported that CCL20 induced EMT via the Erk1/2-Akt pathway [93].

*Therapeutic possibilities:*

As in ovarian cancer, different molecules have been implicated in cervical cancer studies to promote or abrogate specific cytokines or their signalling pathways involved in EMT. TGF-β-mediated EMT changes could be blocked by Hesperetin, a flavonoid in citrus fruits [78]; Epigallocatechin-3-gallate (EGCG), a polyphenolic compound found in green tea [80] and Chalcone L1, which is a natural antioxidant and anti-inflammatory polyphenol sourced from plants [81]. Therefore, Hesperetin/EGCG/Chalcone L1 may have therapeutic benefits as anti-cancer agents for the treatment of cervical cancer. In a study by Li et al. [82] cadherin CDH 20 (belonging to a superfamily of cell-to-cell adhesion molecules) interacted with β-catenin and suppressed TGF-β-mediated EMT in cervical cancer cell lines. This suggests that CDH 20 may act as a tumour suppressor that can inhibit cervical cancer cell migration and invasion and hence may have therapeutic potential. Wu et al. [85] demonstrated that TGF-β1 induced EMT in cervical cancer cells in both Human papillomavirus (HPV)-positive and negative cervical cancer cells. Therefore, TGF-β1 could be used for targeted therapy for cervical cancer irrespective of HPV status. The knockdown of some molecules involved in TGF-β/EMT signalling could halt this EMT pathway and could be used for cancer treatment; such molecules could be CD36, a membrane glycoprotein present on various epithelial cells [83]; RhoE, a RNA/DNA helicase [84]; FAD104, a fibronectin type III domain-containing protein (FNDC) [87]; p68, a type of RNA helicase [89]; and Sine oculis homeobox homolog 1 (SIX1), a transcription factor associated with development but rarely expressed in adults [91]. Hence, targeting CD36, RhoE, FAD104, p68, or SIX alongside TGF-β signalling inhibition could be new therapeutic avenues for cervical cancer treatment.

Zhang et al. [93] reported that the expression of astrocyte-elevated gene-1 (AEG), a multifunctional oncoprotein, was increased by treatment with chemokine CCL20/CCR6. AEG knockdown resulted in abrogation of the EMT changes and disruption of the ERK1/2-Akt signalling induced by CCL20. This implies that AEG is an important component of the CCL20/CCR6-Erk1/2-Akt-EMT pathway and could be a novel targeted therapy for cervical cancer.

**Table 3:** EMT changes associated with cytokines/chemokines in cervical cancer.

Cancer	Cytokine/Chemokine	Mechanism of EMT
Cervical	TGF-β	Epithelial downregulation: E-cadherin [50,78,80,81,82,83,85,86,88,89,90,92,95], ZO-1 [84,87], and β-catenin [82]. Mesenchymal upregulation: Vimentin [80,83,85,88,89,90,92], fibronectin [85,87,95], N-cadherin [78,81,82,86,91], and α-SMA [89]. EMT-TF activation/suppression: *SNAI1* [78,80,83,84], *SNAI2*/Slug [80,83], *TWIST* [78,80,83,86], and *ZEB* [78,80]. Mesenchymal morphological changes: [78,86,88,90,92,95].
	TNF-α	Epithelial downregulation: E-cadherin [86]. Mesenchymal upregulation: N-cadherin [86]. EMT-TF activation/suppression: *TWIST* [86]. Mesenchymal morphological changes: [86].
	IL-6	Epithelial downregulation: E-cadherin [94]. Mesenchymal upregulation: Vimentin [94]. Mesenchymal morphological changes: [94].
	Chemokine CCL20	Mesenchymal upregulation: Vimentin [93], N-cadherin [93], and Matrix metalloproteinase MMP2/MMP9 [93].

### 4.4. Endometrial Cancer/Uterine Cancer

In this systematic review, nine studies on endometrial cancer have been included (one overlapping with ovarian cancer). Various human endometrial cancer cell lines such as HEC-1A, HEC 1B, and Ishikawa cells or human endometrial cancer tissue have been examined for EMT on treatment with various cytokines and chemokines by multiple methods such as immunoblot, immunofluorescence, or quantitative reverse transcriptase PCR (qRT-PCR). Table 4 documents the EMT changes associated with cytokines or chemokines in endometrial cancer. To simplify our findings, the EMT changes in endometrial cancer have been classified under the following sub-headings:

*Mesenchymal morphological changes:*

A change in cell shapes from cobblestone (epithelial) to spindle-like narrow elongated shape (mesenchymal) has been reported in endometrial cancer cells when treated with TGF-β [65,96,97]; autocrine motility factor (AMF), a tumour-secreted cytokine [103]; IL-6 [65]; and chemokine CCL18 [100] and when co-treated with Receptor activator of nuclear factor (RANK)/Receptor activator of nuclear factor kB ligand (RANKL) and chemokine CCL20 [102].

*Downregulation of epithelial markers:*

The studies included have demonstrated a downregulation of epithelial marker E-cadherin in endometrial cancer cells by TGF-β treatment [65,80,96,99], AMF treatment [103], and IL-6 treatment [65] and when treated with chemokine CCL18 [100] and RANK/RANKL/CCL20 [102].

*Upregulation of mesenchymal markers:*

Mesenchymal markers such as vimentin/fibronectin/N-cadherin have been reported to be over-expressed in endometrial cancer when treated with different cytokines and chemokines. Vimentin was reported to be upregulated by treatment with TGF-β [80,96,99], AMF [103], chemokine CCL18 [100], and RANK/RANKL [102]. N-cadherin was over expressed when endometrial cancer cells were treated with TGF-β [65,96,99], IL-6 [65], chemokines CCL18 [100], RANK/RANKL/CCL20 [102], and CXCR4/CXCL12 [101]. α-smooth muscle actin (α-SMA), another mesenchymal marker, was found to be increased in endometrial cancer cells with CXCR4/CXCL12 treatment [101].

*EMT-TF activation/suppression:*

*SNAI1*, *SNAI2*, *TWIST*, and *ZEB* EMT TFs have been reported to be involved in EMT in endometrial cancer cell lines.

*SNAI2*/Slug [80,98] and *SNAI1* [65,80,98] were reported to be upregulated by TGF-β treatment, AMF [103], IL-6 treatment [65], and treatment with RANK/RANKL/CCL20 [102]. *TWIST* has been reported to be upregulated by TGF-β [65,80,96], IL-6 [65], chemokine CCL18 [100], and RANK/RANKL/CCL20 [102], whereas *ZEB* was reported to be upregulated by TGF-β treatment [80].

*Possible pathways and interactions:*

Smad2/3/TGF-β signalling has been reported to be involved in EMT in endometrial cancer cell lines [80]. Chen et al. [96] reported that TGF-β1 possibly induces EMT by the Smad3/*TWIST* signalling pathway in endometrial cancer cells. Li et al. reported that AMF induces EMT in endometrial cancer via the transforming growth factor β receptor 1 (TGFBR1)/ERK/MAPK pathway [103].

*Therapeutic possibilities:*

Chen et al. [96] reported that Isoliqueritigenin (ISL), a flavonoid derived from liquorice and bean sprouts, may be able to reverse TGF-β-induced EMT changes in endometrial cancer cell lines, causing it to be a potential candidate for targeting TGF-β-induced signalling in endometrial cancer. The treatment of an endometrial cancer cell line with fluorene-9-bisphenol (BHPF), a derivative of bisphenol A, in the study by Wang et.al. [98] inhibited the TGF-β1-induced expression of EMT markers, indirectly demonstrating the effect of TGF-β1 on EMT and indicating that BHPF could be used as a possible novel therapy for endometrial cancer.

**Table 4:** EMT changes associated with cytokines/chemokines in endometrial cancer.

Cancer	Cytokine	Mechanism of EMT
Endometrial	TGF-β	Epithelial downregulation: E-cadherin [65,96]. Mesenchymal upregulation: Vimentin [80,96] and N-cadherin [65,96,99]. EMT-TF activation/suppression: *TWIST* [96] and *SNAI2*/Slug [65,80,98]. Mesenchymal morphological changes: [65,96,97].
	IL-6	Epithelial downregulation: E-cadherin [65]. Mesenchymal upregulation: Vimentin and N-cadherin [65]. EMT-TF activation/suppression: *TWIST* [65] and *SNAI1* [65]. Mesenchymal morphological changes: [65].
	AMF	Epithelial downregulation: E-cadherin [103]. Mesenchymal upregulation: Vimentin [103]. Mesenchymal morphological changes: [103].
	RANK/RANKL/CCL20	Epithelial downregulation: E-cadherin [102]. Mesenchymal upregulation: Vimentin [102] and N-cadherin [102]. EMT-TF activation/suppression: *SNAI1*/2 and *TWIST* [102]. Mesenchymal morphological changes: [102].
	CCL18	Epithelial downregulation: E-cadherin [100]. Mesenchymal upregulation: Vimentin [100] and N-cadherin [100]. EMT-TF activation/suppression: *TWIST* [100]. Mesenchymal morphological changes: [100].
	CXCR4/CXCL12	Mesenchymal upregulation: N-cadherin [101] and α-SMA [101].

### 4.5. Risk of Bias in Individual Studies

Each study was evaluated for their methodological integrity, ethical sourcing of materials, and conduct of study before including in the review. The studies that did not clearly state the active compound being investigated or clearly demonstrate in their methodology and results a specific EMT change as a result of treatment with a specific cytokine in gynaecological cancers were excluded from the review. It was not possible to evaluate the quality and methodological soundness of conference abstracts and, hence, they were not included in the study.

## 5. Discussion

Various cytokines and chemokines have been demonstrated to be involved in promoting EMT in gynaecological cancers, such as TGF-β, IL-6, IL-8, and TNF-α. These soluble mediators along with various growth factors such as EGF, fibroblast growth factor (FGF), hepatocyte growth factor (HGF), platelet-derived growth factor (PDGF), tumour growth factor (TGF)-β, and vascular endothelial growth factor (VEGF) along with immune cells in the TME such as the immune infiltrating macrophages, CAFs, neutrophils, and platelets can promote EMT in primary tumours. They can transform epithelial cells to mesenchymal cells by upregulating a variety of EMT transcriptional factors, such as *SNAI1*/*SNAI2*/*ZEB*/*TWIST* that are repressors of epithelial gene such as E-cadherin and activators of mesenchymal genes such as N-cadherin/fibronectin/vimentin, α-smooth muscle actin, etc. Mesenchymal cells invade the surrounding stroma and eventually enter the systemic circulation, reach distant sites and undergo mesenchymal–epithelial transformation (MET), which is essential for the outgrowth of metastases.

Similar to studies on oral SCC [26], NSCLC [29], and breast cancer [30,31], cytokines or chemokines such as interleukins and SDF-1/CXCR4 have been involved in the reduction in the expression of epithelial markers such as E-cadherin in ovarian, endometrial, and cervical cancers and the upregulation of mesenchymal markers such as N-cadherin and/or vimentin. These cytokines regulate EMT via various mechanisms by activating EMT-TFs such as *TWIST*/*SNAI1*/*SNAI2*/*ZEB* and signalling pathways. The predominantly reported EMT signalling pathways among a multitude of pathways and interactions mentioned above involve WNT/β-catenin activation [55] and SMAD2/3 activation by TGF-β [40,41,75] and STAT3 activation by IL-6 [47,48,77]. The roles of cytokines are complicated and they can interact among themselves and with other substances in an inflammatory tumour microenvironment and create a complex permissive milieu of signalling that regulate EMT changes and promote cancer progression and metastasis and also possibly induce drug resistance and allow for the recurrence of cancer.

### Strengths and Limitations of Our Review

The strength of our review is that it presents a comprehensive review of all the English language studies in the last 22 years demonstrating the effect of cytokines/chemokines on EMT in all gynaecological cancers under one platform. However, the use of a specified time range and English language for our search criteria may have introduced a time-period bias and a language bias, respectively. We have performed a grey literature search to reduce the risk of selection bias. The PRISMA 2020 guideline [32] has been used for presenting screened, excluded, and analysed articles in the form of a flow diagram to reduce publication and selection bias. All the studies included used either human tissue or gynaecological cancer cell lines, mentioned the source of all tissues, cell lines, and reagents used and described in detail the tests involved such as immunoblot/immunofluorescence/qRT-PCR.

The limitations are that the articles included are not uniform in their cell type or tissue and modes of experiments. Some studies have used human tissue, while others have used cancer cell lines and some both. Among the cell lines, different sources have been used across the studies included in the systematic review. Due to the different sources/methods/treatment conditions used, it was not possible to synthesise the results using a single statistical test and hence no statistical tests have been used.

## 6. Further Recommendations for Research

We did not find any studies satisfying the inclusion criteria for our systematic review on vulval and vaginal cancers. These cancers are closely anatomically related to cervical cancer and cytokine-mediated EMT has been shown to have a role in cervical cancer progression and metastasis. Hence, further studies are required to investigate the role of cytokines and other soluble factors on EMT in these two cancers. The role of EMT in inducing drug resistance in gynaecological cancers needs further investigation. Additionally, the possibility of halting cancer progression or reversing drug resistance by targeting specific cytokines or their downstream effector molecules and/or EMT markers and EMT-TFs needs extensive exploration.

## 7. Conclusions

Various studies have been included in this systematic review to document the effect of cytokines on EMT in gynaecological cancers, using different cell lines or human tissue from multiple sources. To the best of our knowledge, this study is the only systematic review on the effect of cytokines on EMT in gynaecological cancers. The review summarises all the relevant English language articles on this subject in the last 22 years. However, the studies are not yet exhaustive and further investigations are warranted to explore the roles of cytokines on EMT in gynaecological cancers in depth. EMT is an essential part of cancer progression and, hence, it is possible that targeted therapy affecting these cytokines themselves or either upstream or downstream EMT signalling pathways could be of therapeutic benefit in cancer treatment. Defining the exact role of the cytokines involved and their signalling pathways inducing EMT in a specific type of gynaecological cancer and developing novel targeted therapies could potentially halt cancer progression or metastasis, reduce the risk of cancer recurrence, reverse or prevent drug-resistance in these cancers, and could also supplement available chemotherapy and other therapies appropriately.

## Figures and Tables

**Table 1:** Characteristics of articles included in the systematic review.

Ref No.	Author, Year, and Place	Study Population/Cell Line	Laboratory Methods	Cytokine/Chemokine	Cancer Site	Results	Clinical Implication
[33]	Tian 2022 China	Human ovarian cancer cell line: SKOV3	• Immunoblot	TGF-β	Ovarian	TGF-β treatment of ovarian cancer cells reduced the expression of E-cadherin (*p* < 0.05), whereas it increased vimentin expression. Sorafenib, a pan-protein kinase inhibitor, inhibited the TGF-β effect.	Sorafenib can be used as a therapeutic modality for inhibition of TGF-β-induced EMT in ovarian cancer.
[34]	Yi 2021 China	Human ovarian cancer cell line: SKOV3, Coci, CAOV3, A2780, SW626, HEY, and OVCAR3	• Immunoblot	IL-6	Ovarian	Silencing RIPK4 significantly reduced IL-6 levels (*p* < 0.05) and downregulated vimentin, N-cadherin, and *TWIST* expression but induced an increase in E-cadherin levels. The addition of IL-6 overcame the effects of the silencing of RIPK4 on EMT.	Downregulation of RIPK4 expression can stop EMT by inhibiting IL-6.
[35]	Sicard 2021 Canada	Human ovarian cancer cell line: chemo sensitive ES-2 and chemo-resistant SKOV-3	• Immunoblot • Immunofluorescence	TGF-β	Ovarian	TGF-β treatment of ovarian cancer cells increased vimentin, fibronectin, Snail, Slug, and Smad-3 expression in ES-2 cells (chemo-sensitive) but not in SKOV-3 cells (chemo-resistant).	Benefit of targeting TGF-β signalling to prevent EMT may be limited by chemo-sensitiveness of the cancer.
[36]	Ren 2021 China	Human ovarian cancer cell line: OVCAR3	• Immunoblot • RT-PCR Immunohistochemistry	TGF-β	Ovarian	TGF-β-treated ovarian cancer cells demonstrated increased miR-200a level, decreased sex-determining region Y-box 4 (SOX4) level, and promoted EMT changes such as reduction in E-cadherin and induction of vimentin and N-cadherin.	miRNA-200a/TGF-β/SOX4 signalling is involved with EMT in ovarian cancer and targeting this pathway may help reverse EMT changes.
[37]	Wen 2020 China	Human ovarian cancer cell line: SKOV3, A2780	• Immunoblot	IL-8	Ovarian	E-cadherin level was markedly decreased and the vimentin level was markedly increased in the exogenous IL-8–treated group with elevation of Wnt/β-catenin signalling, the changes being blocked by using IL-8 inhibitor, Reparixin.	IL-8 and IL-8 receptors CXCR1 and CXCR2 could be potentially used as bio-markers and therapeutic targets.
[38]	Guo China 2020	Human ovarian cancer cell line: SKOV3	• Immunoblot	IL-17	Ovarian	E-cadherin level was significantly decreased and the vimentin, N-cadherin, Snail, Slug, and *TWIST* levels were markedly increased with IL-17 treatment possibly via the expression of metastasis-associated genes-1 (MTA1).	IL-17/MTA-1 axis as targeted therapy.
[39]	Ameri 2019 Qatar	Human ovarian cancer cell line: PA-1, SKOV3, SW626, CAOV3, OVCAR3, APOCC, A2780, and A2780 CIS (cisplatin resistant)	• IHC • Immunofluorescence	TGF-β	Ovarian	TGF-β treatment activates EMT partially by upregulating some EMT signature genes, such as matrix metalloproteinase (MMP2), FN1, and VIM, and downregulating E-cadherin. TGF-receptor kinase I inhibitor (LY364947) can revert the TGF-β-mediated effect.	Targeting TGF-β signalling for treatment of ovarian cancer possibly by using TGF-receptor kinase I inhibitor (LY364947).
[40]	Bai 2019 China	Human ovarian cancer cell line: SKOV3	• IHC • Immunoblot	TGF-β	Ovarian	FXYD domain-containing ion transport regulator 5 (FXYD5) potentiated TGF-β/SMADs signalling and TGF-β-induced EMT as demonstrated by the rise in vimentin, N-cadherin, *SNAI2*, and MMP 2 and 9 (mesenchymal markers) and the reduction in β-catenin (epithelial marker).	FXYD5 could be targeted to breakdown TGF-β/SMADs signalling.
[41]	Guo 2019 China	Human ovarian cancer cell line: SK-OV-3 and OVCAR-3 Human ovarian cancer tissue	• qRT-PCR • Immunoblot • IHC	TGF-β	Ovarian	Platelet-derived TGF-β resulted in upregulated mesenchymal markers (Snail, vimentin, N-cadherin, fibronectin-1, and matrix metalloproteinase-2), downregulated E-cadherin and increased phosphorylation of Smad2. TβR I inhibitor A83–01 reversed the EMT-like alterations.	Targeting TGF-β signalling for treatment of ovarian cancer possibly by using TβR I inhibitor A83–01.
[42]	Wang 2019 China	Human ovarian cancer cell line: SKOV3, HO8910	• Immunofluorescence • Immunoblot	Bone morphogenetic protein (BMP)-9	Ovarian	BMP9 treatment downregulated the expression of E-cadherin and upregulated the expression of N-cadherin, *SNAI1, SNAI2*, and *TWIST* in a dose-dependent manner.	BMP9 may be useful as a new targeted treatment option.
[43]	Bhattacharya 2018 India	Human ovarian cancer cell line: SKOV3, PA1	• IHC • Immunofluorescence • Immunoblot	TGF-β	Ovarian	TGF-β1 treatment of ovarian cancer cell lines demonstrated the upregulation of *ZEB1*/*2* and *SNAI1*/*2*, reduction in E-cadherin and Claudin 7, and increase in N-cadherin and vimentin.	Targeting TGF-β signalling for treatment of ovarian cancer.
[44]	Mitra 2018 India	Human ovarian cancer cell line: SKOV3, PA1	• IHC • Immunofluorescence • Immunoblot	TGF-β	Ovarian	TGF-β1 treatment of ovarian cancer cell lines demonstrated upregulation of *ZEB1* and *ZEB1* knockdown resulted in reduced expression of vimentin.	Targeting TGF-β/*ZEB1* signalling for treatment of ovarian cancer.
[45]	Hong 2018 China	Human ovarian cancer tissue	• IHC • Immunofluorescence • Immunoblot	TGF-β	Ovarian	Inhibition of TGF-β expression (by SiRNA/LKB1) suppressed the expression levels of EMT markers *SNAI2*, *TWIST*, and *ZEB1*.	LKB1 activation may be used to inhibit the TGF-β-mediated EMT pathway.
[46]	Matsumoto 2018 Japan	Human ovarian cancer cell line: TOV-21G	• qRT-PCR • Immunoblot	LEFTY TGF-β	Ovarian	Left–right determination factor (LEFTY), member of TGF-β superfamily, promoted spindle-like cell shape, reduction in E-cadherin expression, increase in N-cadherin, vimentin, and *SNAI1* expression.	Targeting TGF-β/LEFTY/Smad signalling for treatment of ovarian clear cell carcinoma.
[47]	Wang 2018 China	Human ovarian cancer cell line: OVCAR3	• IHC • Immunofluorescence • Immunoblot	IL-6	Ovarian	Cancer-associated fibroblasts (CAFs) increased IL-6 secretion that upregulated N-cadherin and vimentin and decreased the expression of E-cadherin in ovarian cancer cells via the JAK2/STAT3 pathway.	CAFs may constitute new therapeutic targets as they release cytokines in the TME that potentiate EMT.
[48]	Ma 2018 China	Human ovarian cancer cell line SKOV3 Human ovarian cancer tissue	• IHC • Immunofluorescence	TGF-β	Ovarian	TGF-β mediates CD146 expression and E-cadherin to N-cadherin switch in ovarian cancer cells. STAT3/*TWIST* pathway is involved in E-cadherin downregulation and ERK pathway in N-cadherin upregulation.	Co-targeting CD146 and TGF-β may have better response in ovarian cancer treatment.
[49]	Wu 2018 China	Human ovarian cancer cell line SKOV3, A2780	• Immunofluorescence	TGF-β	Ovarian	TGF-β treatment in ovarian cancer cell lines led to increased ST3GAL1 expression and lower levels of epithelial marker E-cadherin and increased expression of N-cadherin and vimentin and the EMT effect was reversed with knocking down ST3GAL1 expression.	ST3GAL1 may be a promising target for overcoming drug resistance in ovarian cancer treatment.
[50]	Liu 2018 China	Human ovarian cancer cell line SKOV3	• Immunoblot	TGF-β	Ovarian	TGF-β treatment, 5 ng/mL for 48 h, increased expression of N-cadherin and reduced E-cadherin expression. ATP-binding cassette (ABC) transporter 7 (ABCA7) potentiated TGF-β-induced EMT.	TGF-β1/ABCA7 could be targets for prevention of ovarian cancer.
[51]	Li 2018 China	Human ovarian cancer cell line OVCAR3	• Immunoblot	TGF-β TNF-α	Ovarian	TGF-β and/or TNF-α treatment, increased expression of N-cadherin and reduction in E-cadherin in OVCAR cells and caused mesenchymal spindle phenotype. These changes were blocked by Chrysin, a bioactive flavonoid.	Chrysin could have therapeutic benefits in blocking TGF-β/TNF-α signalling.
[52]	Zheng 2018 China	Human ovarian cancer cell line IGROV-1 and HO-8910	• Immunoblot	CXCL12- CXCR4/CXCR7	Ovarian	CXCL12 induced mesenchymal morphological changes including spindle-like cell morphology, podia and stress fiber formation, a decreased E-cadherin expression, and increased mesenchymal N-cadherin and vimentin expressions. In addition, the protein levels of *ZEB1*, *ZEB2*, *TWIST1*, β-catenin, MMP-2, and MMP-9 were significantly increased. These effects were countered by CXCR4 antagonist AMD3100, but not by the anti-CXCR7 antibody.	CXCL12-CXCR4 chemokine axis represents a potential therapeutic strategy.
[53]	Figueras 2018 Spain	Human ovarian cancer cell line SKOV3	• IHC • Immunoblot	CXCR4	Ovarian	CXCR4-positive cells expressed low levels of CDH-1 and E-cadherin (epithelial markers) and high levels of the mesenchymal markers *ZEB1*, *ZEB2*, and *SNAI1* compared with CXCR4-negative cells, possibly by Src activation.	CXCR4 and Src inhibitors may be therapeutic agents for serous ovarian cancer.
[54]	Pazos 2017 Argentina	Human ovarian cancer cell line SKOV3, IGROV1	• Immunoblot	TGF-β	Ovarian	TGF-β treatment led to increased expression of N-cadherin and reduction in E-cadherin and increased expression of the transcription factors Snail, Slug, *TWIST*, and *ZEB1*. γ-secretase inhibitor blocked TGF-β-induced EMT.	γ-secretase inhibitor could be a novel therapeutic agent.
[55]	Mitra 2017 India	Human ovarian cancer cell line SKOV3, OAW-42	• Immunoblot • qRT-PCR • Immunofluorescence	TGF-β	Ovarian	Upregulation of mesenchymal markers N-cadherin and vimentin with downregulation of the epithelial markers E-cadherin and claudin were observed with TGF-β treatment alongside Wnt signalling activation. Inhibition of the TGF-β pathway by the receptor inhibitor (TGF-βRI) led to the downregulation of vimentin and inhibition of the Wnt signalling pathway and downregulated N-cadherin.	Targeting TGF-β signalling for treatment of ovarian cancer.
[56]	Norita 2017 Tokyo	Human ovarian cancer cell line SKOV3	• Immunoblot • qRT-PCR	TGF-β	Ovarian	Knockdown of Vasohibin2 (VASH2) in cancer cells abrogated the TGF-β1-induced reduced expression of epithelial marker E-cadherin and the elevated expression of mesenchymal markers MMP2, fibronectin, *ZEB2*, and *SNAI2*, suggesting that endogenous VASH2 is required for TGF-β1-induced EMT.	VASH2 could be a novel molecular target for the prevention of EMT in ovarian cancer.
[57]	Powell 2016 USA	Human ovarian cancer cell line SKOV3, HEY	• Immunoblot	TGF-β	Ovarian	TGF-β treatment induced fibronectin expression. Heat shock protein1 (HSF-1) knockdown led to reduced TGF-β-mediated fibronectin expression.	TGF-β/HSP1 signalling could be used as co-targets for ovarian cancer treatment.
[58]	Hou 2016 China	Human ovarian cancer cell line SKOV-3 Cells	• Immunoblot • qRT-PCR	TGF-β	Ovarian	TGF-β treatment (10 ng/mL) for 24 h led to cell morphology changes from pebble to spindle and elongated mesenchymal features, decreased expression of E-cadherin, and increased vimentin, *SNAI2*, *SNAI1*, and β-catenin (*p* < 0.05). 1α,25(OH)2D3 abrogated these effects.	TGF-β signalling could be targeted by using 1α,25(OH)2D3 as a novel therapeutic agent.
[59]	Zhou 2016 Canada	Human ovarian cancer cell line SKOV3, OVCA429	• Immunoblot	TGF-β	Ovarian	Notch1 activation upregulated the expression of TGF-β and upregulated TGF-β/Smad signalling, which causes EMT, and, on the other hand, TGFβ increased the expression of Notch ligand, Jagged1, in EOC cells.	NOTCH/TGF-β as novel therapeutic targets for treatment of ovarian cancer.
[60]	Zhang 2016 China	Human ovarian cancer cell line SKOV3	• Immunoblot	TGF-β	Ovarian	TGF-β treatment led to increased expression of N-cadherin and reduction in E-cadherin. TGF-β induced expression of Hematopoietic Pre-B-cell leukaemia transcription factor (PBX)-interacting protein (HPIP). The knockdown of HPIP abrogated this effect of TGF-β.	Targeting HPIP could be a new therapeutic option for inhibiting TGF-β signalling.
[61]	Ye 2016 China	Human ovarian cancer cell line: SKOV3, 3AO	• Immunoblot	TGF-β	Ovarian	TGF-β caused E-cadherin downregulation and N-cadherin, vimentin, and Snail upregulation. Overexpression of TET3 reversed TGF-β1-induced EMT changes.	TGF-β1-TET3-miR-30d signalling axis could be useful for epigenetic regulation of EMT changes.
[62]	Cardenas 2016 USA	Human ovarian cancer cell line: SKOV3	• Immunoblot	TGF-β	Ovarian	TGF-β caused cell morphology change from epithelial to mesenchymal, E-cadherin downregulation, vimentin upregulation, and upregulation of *SNAI1, SNAI2*, and *ZEB*.	TGF-β1 signalling could be targeted for treatment of ovarian cancer.
[63]	Zhou 2016 Canada	Human ovarian cancer cell line: SKOV3	• Immunoblot	Growth differentiation factor 8 (GDF-8)	Ovarian	GDF-8 caused E-cadherin downregulation but had no effect on N-cadherin and upregulated *SNAI1* and *SNAI2*. GDF-8 worked via activin receptor-like kinase (ALK) 4 and 5 pathways.	GDF-8/ALK signalling could be targeted for ovarian cancer treatment.
[64]	Ye 2015 China	Human ovarian cancer cell line: SKOV3, 3AO	• Immunoblot	TGF-β	Ovarian	TGF-β caused E-cadherin downregulation and N-cadherin, vimentin, and *SNAI1* upregulation. Overexpression of miR-30d (micro-RNA) blocked TGF-β1-induced EMT changes.	Targeting the TGF-β1-miR-30d signalling axis for epigenetic regulation of EMT changes.
[65]	So 2015 Republic of Korea	Human ovarian cancer cell line: SKOV-3 and IGROV-1	• qRT-PCR • Immunoblot • Immunofluorescence	IL-6 TGF-β1	Ovarian	Cell morphology showed changes related to EMT-cell dissociation and fibroblastic morphologic changes with IL-6 and TGF-β1 treatment. IL-6 and TGF-β1-treated cancer cells had increased *SNAI1*, *TWIST*, and N-cadherin expression and decreased E-cadherin expression compared with untreated cancer cells.	IL-6 and TGF-β1 could be targeted for cancer therapy and the prevention of progression.
[66]	Yin 2015 China	Human ovarian cancer cell line: SKOV3, OVCAR3	• Immunoblot	IL-8	Ovarian	E-cadherin was decreased, while β-catenin was elevated in IL-8 pre-treated cells.	IL-8 could be used as a biomarker or a target for ovarian cancer treatment.
[67]	Long 2015 China	Human ovarian cancer cell line A2780, SKOV3	• IHC • Immunofluorescence	Chemokine (C-C motif) ligand 5 (CCL5) and its receptors chemokine (C-C motif) receptor (CCR) 1/3/5	Ovarian	Paracrine CCL5 from ovarian cancer stem-like cells (CSLC) activates the NF-κB signalling pathway in ovarian non-CSLCs via binding CCR1/3/5, thereby inducing EMT (decreased E-cadherin and increased mesenchymal markers vimentin and Snail). Anti-CCL5 antibody inhibited the CSLC-induced increase in expression of the mesenchymal marker vimentin and EMT-TFs *SNAI1* and *SNAI2* (*p* < 0.05). Anti-CCL5 antibody inhibited CSLC-induced EMT.	Targeting the CCL5-CCR1/3/5-NF-κB pathway could be an effective strategy to prevent ovarian cancer metastasis.
[68]	Yu 2015 China	Human ovarian cancer cell line SKOV3	• Immunoblot	Chemokine CCL19/CCR7	Ovarian	CCL19 induced AKT and ERK phosphorylation and upregulated the expression of vimentin, *SNAI1*, and N-cadherin and downregulated the expression of E-cadherin. CXCR7 siRNA significantly abrogated these effects of CCL19.	Using CXCR7 antagonist could be possible treatment modality for ovarian cancer.
[69]	Cheng 2015 China	Human ovarian cancer cell line H08910, A2780, SKOV-3, ES-2 and HEY	• IHC • Immunofluorescence • Immunoblot	Chemokine CCL19/CCR7	Ovarian	CCL19 treatment upregulated p-CrkL, p-AKT, p-ERK, and EMT markers (N-cadherin, *SNAI1*, and MMP9) in SKOV-3 cells and downregulated E-cadherin expression. Blocking CrkL reduced CCL19-stimulated ERK signalling and EMT.	Blocking CrkL and CCL19 pathways may be of more benefit than blocking either pathway alone.
[70]	Teng 2014 China	Human ovarian cancer cell line SKOV3, 3AO	• Immunoblot	TGF-β	Ovarian	TGFβ1 treatment decreased the expression of E-cadherin and increased expression of vimentin and N-cadherin alongside an increase in expression of a protein called Inhibitor-of-differentiation (Id-1). Blocking Id-1 halted the TGF-β mediated effect on EMT	Targeting Id-1 protein could be a new therapeutic option.
[71]	Cardenas 2014 USA	Human ovarian cancer cell line SKOV3	• Immunoblot	TGF-β	Ovarian	TGFβ1 treatment led to distinct morphological changes as cells changed from the cobblestone appearance characteristic of epithelial cells to the fusiform shape indicative of a mesenchymal phenotype. Additionally, there was a decreased expression of E-cadherin and increased vimentin expression. TGF-β induced DNA methyltransferases (DNMT) and treatment with DNMT inhibitor (SGI-110) prevented TGF-β induced EMT.	Targeting DNMT may reverse the EMT changes or EMT gene suppressions caused by TGF-β induced DNA methylation in ovarian cancer.
[72]	Lin 2014 China	Human ovarian cancer cell lines: SKOV-3, and PC-3	• Immunoblot	TGF-β	Ovarian	TGFβ1 treatment (5 ng/mL) led to decreased expression of E-cadherin and increased expression of *SNAI1*. Treatment with serial concentrations of BEZ235 (a dual PI3K/ mTOR inhibitor that is currently in phase 1/2 clinical trials) reversed the effects of TGF-β.	Targeting TGF-β downstream signalling (PI3K/mTOR) for treatment of ovarian cancer.
[73]	Chen 2014 USA/China	Human ovarian cancer cell lines: SKOV-3 and OVCAR3	• Immunoblot	TGF-β	Ovarian	TGFβ1 treatment led to decreased expression of E-cadherin and increased expression of *SNAI2* and vimentin. Krüppel-like factor 4 (KLF4) expression significantly inhibited TGF-β-induced EMT.	KLF4 functions as a tumour suppressor and can attenuate TGF-β-induced EMT.
[74]	Cheng 2012 Canada	Human ovarian cancer cell lines: Serous borderline ovarian tumour (SBOT), Low-grade ovarian cancer (MPSC1)	• Immunoblot	TGF-β	Ovarian	TGFβ1 treatment decreased the expression of E-cadherin and increased expression of N-cadherin alongside upregulation of the transcriptional repressors of E-cadherin, *SNAI1, SNAI2, TWIST*, and *ZEB1* in serous borderline ovarian tumour (SBOT) cells. These effects could be reversed by TGF-β receptor 1 (TbRI) depletion.	Targeting TGF-β signalling for prevention of progress of ovarian cancer.
[75]	Cao 2012 USA	Human ovarian cancer cell line SKOV3	• qRT-PCR • IHC • Immunoblot	TGF-β	Ovarian	TGF-β1-induced tissue transglutaminase expression in TGF-β1-treated SKOV3 cells, which underwent characteristic morphological changes of EMT, from a compact shape to an elongated, dispersed phenotype, and demonstrated a reduction in E-cadherin expression, associated with increased expression of N-cadherin, *ZEB1*, and Tissue transglutaminase 2 (TG 2) and SMAD2/3 signalling activation, all of which were blocked by SD208 (TGF-β receptor I serine threonine kinase inhibitor) and also by TG2 blocking.	Targeting TG2/TGF-β signalling/dual targeting for treatment of ovarian cancer.
[76]	Xu 2010 Canada	Human epithelial ovarian cancer (EOC) cell line OVCA429	• Immunoblot	TGF-β	Ovarian	TGF-β and Epidermal growth factor (EGF) signalling pathways synergistically induce EMT and render EOC cells a more invasive phenotype. They induced transcription repressors *SNAI1* and *SNAI2*, repressed E-cadherin, and increased MMP-2 expression.	Simultaneous targeting of both TGF-β and EGF signalling pathways for prevention of spread of ovarian cancer.
[77]	Colomiere 2009 Australia	Human ovarian cancer cell lines OVCA 433 and SKOV3	• IHC	IL-6	Ovarian	Co-culture of ovarian cancer cell lines with Epidermal growth factor (EGFR) led to activation of IL-6 receptor (IL-6 R), STAT3 signalling, upregulation of N-cadherin, vimentin, and nuclear translocation of β-catenin.	Blocking EGFR/IL-6R/STAT3 signalling may prevent ovarian cancer progression.
[78]	Wang 2022 China	Human cervical cancer cell lines: HeLa, CaSki	• Immunoblot • Immunofluorescence	TGF-β	Cervical	TGF-β1 incubation (10 ng/mL; 24 h) led to spindle-like mesenchymal phenotype, upregulation of N-cadherin, and down regulation of E-cadherin (*p* < 0.001), upregulation of *SNAI1*, *TWIST2*, and *ZEB*, which could be reversed by administration of Hesperetin (10 and 20 μM/L), a flavonoid in citrus fruits.	Potential benefits of hesperetin as a therapeutic modality for treatment of cervical cancer.
[79]	Zuo 2022 China	Human cervical cancer cell line: CaSki	• Immunoblot • qRT-PCR	IL-9	Cervical	IL-9-treated cells had reduced expression of N-cadherin and vimentin and increased expression of E-cadherin (*p* < 0.05).	IL-9 has anti-tumour effect and could be beneficial in cervical cancer treatment.
[80]	Panji 2021 Iran	Human cervical cancer cell lines: HeLa, SiHa	• Immunoblot • qRT-PCR	TGF-β	Cervical	TGF-β1 incubation (10 ng/mL for 24 h) led to downregulation of E-cadherin (*p* < 0.05), while vimentin was significantly upregulated (*p* < 0.05), accompanied with increased expression of Snail (*p* < 0.01), *ZEB* (*p* < 0.01), *TWIST* (*p* < 0.01), *SNAI2* (*p* < 0.05), and TGF-β signalling molecules Smad 2 and 3. Pre-incubation with TGFβRI/II inhibitor and green tea extract returned all the EMT markers to their prior levels (*p* < 0.05).	Potential benefits of green tea in halting TGF-β-induced EMT.
[81]	Kuruc 2021 Slovakia	Human cervical cancer cell lines: HeLa	• Immunoblot	TGF-β	Cervical	TGF-β treatment significantly downregulated E-cadherin and upregulated N-cadherin expression in HeLa cells. Treatment with Chalcone L1 reversed this effect.	Potential benefits of Chalcone L1 in abrogating TGF-β-induced EMT.
[82]	Li 2020 China	Human cervical cancer cell lines: SiHa and CaSki Human cervical cancer tissue (n = 48)	• Immunoblot • qRT-PCR	TGF-β	Cervical	TGF-β1 treatment notably reduced E-cadherin and β-catenin while increasing N-cadherin expression and increased the levels of pSmad2/3. Addition of CDH20 seemed to negate these effects of TGF-β.	Targeting TGF-β signalling for treating cervical cancer. CDH20 may have therapeutic potential.
[83]	Deng 2019 China	Human cervical cancer cell lines: C33a, Hce1, HeLa, and SiHa Human cervical cancer tissue (n = 133) and control normal cervical tissue (n = 47)	• Immunoblot • Immunofluorescence	TGF-β	Cervical	TGF-β treatment reduced E-cadherin expression and enhanced the expression levels of CD36, vimentin, *SNAI1*, *SNAI2*, and *TWIST* suggesting a possible synergy between TGF-β and CD 36 in promoting EMT.	CD36 could be an effective treatment target alongside TGF-β.
[84]	Nishizuka 2019 Japan	Human cervical cancer cell lines: HeLa	• Immunoblot • qRT-PCR	TGF-β	Cervical	TGF-β1 treatment decreased the expression level of ZO-1, an epithelial marker, whereas it increased the expression of fibronectin, alongside elevated Rho-E expression. RhoE knockdown led to increased levels of fibronectin and Snail and reduced ZO-1 expression. This suggested that reduction in RhoE expression enhanced TGF-β-induced EMT. RhoE is a negative regulator of TGF-β-mediated EMT signalling.	Targeting RhoE signalling may be explored as a treatment option.
[85]	Wu 2017 China	Human cervical cancer cell lines: SiHa, C33a	• Immunoblot • Immunofluorescence	TGF-β	Cervical	A significant reduction in E-cadherin and an increase in vimentin and fibronectin were induced in TGF-β1 treated both HPV-positive and -negative cells.	TGF-β1 can be targeted for cervical cancer therapy irrespective of HPV status.
[86]	Dong 2017 China	Human cervical cancer cell lines: HeLa	• Immunoblot • qRT-PCR	TNF-α TGF-β	Cervical	TNF-α- and TGF-β-induced mesenchymal morphological changes from cobblestone-like to spindle-like shape in HeLa cells either alone or with combination treatment. TGF-β and/or TNF-α resulted in E-cadherin downregulation and N-cadherin upregulation and increased NF-κB and *TWIST1* levels. NF-κB or *TWIST1* knockdown reversed the EMT changes.	Targeting TNF-α or TGF-β signalling with NF-κB/*TWIST1* knockdown may be a possible approach for treatment of cervical carcinoma.
[87]	Goto 2017 Japan	Human cervical cancer cell lines: HeLa, CaSki	• Immunoblot • qRT-PCR	TGF-β	Cervical	TGF-β1 treatment decreased the expression level of ZO-1, an epithelial marker, whereas it increased the expression of fibronectin, especially in Fad-104 knockdown cells. Furthermore, expression of TFs *SNAI1* and *SNAI2* were elevated in Fad104 knockdown cells treated with TGF-β1.	Targeting TGF-β/Fad 104 signalling could have therapeutic benefits.
[88]	Cheng 2017 China	Human cervical cancer cell lines: HeLa, SiHa	• Immunoblot • qRT-PCR	TGF-β	Cervical	TGF-β treatment reduced E-cadherin expression significantly and increased vimentin, Pyruvate kinase M2 (PKM2), and mTOR expression. Rapamycin (mTOR inhibitor) blocked mTOR pathway and abolished TGF-β1-induced EMT, reduced mTOR/p70s6k signalling, and downregulated PKM2 expression.	Inhibition of mTOR/p70s6k/PKM2 signalling could be a new therapeutic avenue for cervical cancer treatment.
[89]	Li 2017 China	Human cervical cancer cell lines: CaSki	• Immunoblot • qRT-PCR	TGF-β	Cervical	TGF-β treatment induced mesenchymal morphological changes in cervical cancer cells and reduced E-cadherin expression and increased vimentin, fibronectin, SMA, and Smad2 signalling. These effects were partially revered by p68 knockdown (p68 belongs to a group of RNA helicase).	p68/TGF-β1 signalling inhibition could have therapeutic effect.
[90]	Cheng 2016 China	Human cervical cancer cell lines: HeLa, SiHa	• Immunoblot • qRT-PCR	TGF-β	Cervical	TGF-β treatment reduced E-cadherin expression significantly and increased vimentin expression. Metformin reversed TGF-β-induced EMT acting via blocking m-TOR signalling.	Metformin as a form of targeted therapy to inhibit TGF-β EMT signalling.
[91]	Sun 2016 China	Human cervical cancer cell lines: SiHa	• Immunoblot • qRT-PCR	TGF-β	Cervical	TGF-β treatment slightly suppressed E-cadherin expression and negligibly induced N-cadherin expression. These effects of TGF-β were supplemented and enhanced by addition of Sine oculis homeobox homolog 1 (SIX1), a transcription factor associated with development.	Targeting TGF-β/SIX signalling for treatment of cervical cancer.
[92]	He 2015 China	Human cervical cancer cell lines: SiHa	• Immunoblot • Immunofluorescence staining	TGF-β	Cervical	48 h of TGF-β (10 ng/mL) treatment induced mesenchymal spindle shape, reduced E-cadherin expression significantly, and increased vimentin expression.	Targeting TGF-β signalling for halting EMT changes in cervical cancer.
[93]	Zhang 2015 China	Human cervical cancer cell lines: SiHa Human cervical cancer tissue (n = 94) and eight paired cancer and normal cervical tissue	• Immunoblot • IHC	Chemokine (C-C motif) ligand 20 (CCL20) Chemokine (C-C motif) receptor 6 (CCR6)	Cervical	CCL20/ CCR6 treatment led to a dose-dependent increase in expression of mesenchymal markers—vimentin, N-cadherin, and MMP2 as well as the expression of astrocyte elevated gene-1 (AEG-1), pERK1/2, and pAkt. The AEG knockdown resulted in abrogation of the EMT changes and ERK1/2-Akt signalling.	CCL20/CCR6–AEG-1– EMT pathway could be a possible target for cervical cancer treatment.
[94]	Miao 2014 China	Human cervical cancer cell lines: C33a, HeLa Human cervical cancer tissue and control normal cervical tissue	• Immunoblot • Immunofluorescence • qRT-PCR	IL-6	Cervical	IL-6 treatment induced cell elongation and increased scattering conforming to mesenchymal cell morphology. Cervical cancer cells treated with IL-6 for 48 h decreased E-Cadherin expression but significantly increased vimentin expression.	Blocking IL6/STAT3 pathway may be a potential approach to treat cervical carcinoma.
[95]	Yi 2001 Korea/ USA	Human cervical cancer cell lines: SiHa	• Immunofluorescence • Immunoprecipitation	TGF-β1	Cervical	Treatment with 10 ng/mL of TGF-β1 caused mesenchymal changes—such as elongated fibroblastic shape, the cells being 8.5 times longer, and caused re-organisation of the actin cytoskeleton. Additionally, raised fibronectin level and reduced E-cadherin expression were found; these effects were not completely reversed by TGF-β removal/addition of TGF-β blocking antibodies.	Targeting TGF-β signalling for halting/slowing EMT changes in cervical cancer, even if not completely reversible.
[96]	Chen 2021 Taiwan	Human endometrial cancer cell lines: HEC-1A Ishikawa	• Immunoblot • Immunofluorescence	TGF-β1	Endometrial	TGF-β1 treatment (10 ng/mL) resulted in mesenchymal spindle-like shape of endometrial cancer cells. TGF-β1 suppressed the expression of E-cadherin and enhanced the expression of N-cadherin and vimentin. Isoliqueritigenin (ISL) inhibited TGF-β1-induced EMT changes.	ISL may have potential benefit in treatment of metastatic endometrial cancer.
[97]	Zhang 2020 China	Human endometrial cancer cell lines: HEC-1A	• Immunoblot	TGF-β1	Endometrial	TGF-β1 treatment (10 ng/mL) resulted in fibroblast-like features in HEC-1A cells as opposed to a cobblestone-like appearance in control cell lines. TGF-β1 suppressed the expression of E-cadherin and enhanced the expression of the mesenchymal markers Snail and α-SMA. miR-320a and miR-340-5p inhibited TGF-β1 induced EMT.	miR-320a and miR-340-5p as treatment options to target TGF-β-induced EMT.
[98]	Wang 2019 China	Human endometrial cancer cell line: Ishikawa	• Immunoblot	TGF-β1	Endometrial	Fluorene-9-bisphenol (BHPF) significantly inhibited the EMT process of Ishikawa cells by blocking transforming growth factor-β (TGF-β) signalling pathway, more specifically by reducing the downstream proteins of TGF-β pathway, p-Smad2/3, and *SNAI2*.	BHPF could be used to prevent TGF-β-mediated EMT in endometrial cancer.
[99]	Wang 2018 China	Human endometrial tissue (from endometrial cancer and adjoining non-cancer area)	• Immunoblot • IHC	TGF-β1	Endometrial	Treatment with CAF resulted in increase in TGF-β in culture medium and was associated with significantly increased N-cadherin and vimentin expressions and reduced E-cadherin expression (*p* < 0.05).	Targeting TGF-β signalling for treatment of endometrial cancer.
[100]	Jing 2018 China	Endometrial cancer specimen—86 Normal endometrial tissue—85 Human endometrial cancer cell line: Ishikawa	• IHC • Immunoblot • qRT-PCR	Chemokine Ligand 18 (CCL18)	Endometrial	M2 macrophages treated with ER-α agonist-induced EMT in Ishikawa cells via CCL18 using the KIF5B pathway; this effect was reversed by anti-CCL18 neutralising antibody.	CCL-18 and KIF pathways could be potential targets for endometrial cancer treatment.
[101]	Ding 2017 Taiwan	Human endometrial cancer cell lines: HEC-1A, Rl-95	• Immunoblot • qRT-PCR	CXCR4/CXCL12	Endometrial	CXCR4/CXCL12 induced N-cadherin and α-smooth muscle actin (α-SMA) in endometrial cancer cell lines and these could be blocked by a neutralizing antibody specific to either CXCL12 or CXCR4.	CXCR4/CXCL12 could be targeted for treatment of endometrial cancer.
[102]	Liu 2016 China	Endometrial cancer tissue of different stages (I–III) Human endometrial cancer cell lines: HEC-1A and Ishikawa cells	• Immunofluorescence • qRT-PCR • Immunoblot	Receptor activator of nuclear factor (RANK)/Receptor activator of nuclear factor kB ligand (RANKL) Chemokine ligand 20 (CCL20)	Endometrial	RANK level positively correlates with N-cadherin (*p* = 0.0229) and vimentin (*p* = 0.0398), but negatively with E-cadherin (*p* = 0.0118), so RANK/RANKL activation initiates EMT in endometrial cancer cells. RANK/RANKL promotes the expression and secretion of chemokine CCL20, which in turn facilitates EMT in RANK over-expressed endometrial cancer cells.	RANK/RANKL/CCL20 pathway could be a promising target for preventing metastasis and progression in endometrial cancer.
[65]	So 2015 Republic of Korea	Human endometrial cancer cell lines: Ishikawa	• qRT-PCR • Immunoblot • Immunofluorescence	IL-6 TGF-β1	Endometrial	Endometrial cells treated with IL-6 and TGF-β1 showed cell dissociation and fibroblastic morphologic changes consistent with EMT. IL-6- and TGF-β1-treated cancer cells had significantly increased *SNAI1*, *TWIST*, and N-cadherin expression and decreased E-cadherin expression compared to untreated cancer cells.	IL-6 and TGF-β1 could be targeted for cancer therapy and prevention of progression of endometrial cancer.
[103]	Li 2015 China	Human endometrial cancer cell lines: Ishikawa and HEC-1B	• IHC • qRT-PCR • Immunoblot	Autocrine motility factor (AMF)	Endometrial	AMF levels positively correlated with vimentin (*p* = 0.012) and *SNAI1* (*p* = 0.021) levels, inversely correlated with the levels of E-cadherin (*p* = 0.035). On AMF silencing, endometrial cancer cell morphology reverted from mesenchymal to epithelial, E-cadherin expression increased and vimentin signal weakened along with downregulation of *SNAI1* and transforming growth factor β receptor 1 (TGFBR1) and reduced p-ERK1/2 of MAPK pathway.	AMF/MAPK EMT signalling might be a potential target for prevention of spread and treatment of endometrial cancer.

## Data Availability

Not applicable, as review article data were derived from other studies that have been mentioned using references.

## Supplementary Materials

The following supporting information can be downloaded at: https://www.mdpi.com/article/10.3390/cells12030416/s1, File S1: Detailed search criteria for the systematic review according to PRISMA guidelines.
