# Supplementary material for: The Role of Cytokines in Epithelial–Mesenchymal Transition in Gynaecological Cancers: A Systematic Review
Source: Cells. 2023 Jan 26;12(3):416. doi: 10.3390/cells12030416 (PMC9913821; doi:10.3390/cells12030416)
Supplement: Supplementary file 1 [file cells-12-00416-s001.zip › cells-2022839-supplementary.pdf]

cells

MDPI

File S1: Detailed search criteria for the systematic review according to PRISMA guidelines.

Systematic review

The role of cytokines in epithelial-mesenchymal transition in gynaecological cancers: a systematic review

Irene Ray [1,2], Agnieszka Michael [1,2], Lisiane B. Meira [1], Patricia E. Ellis [2]

File S1: Detailed search criteria for the systematic review according to PRISMA guide-lines.

Embase <1974 to 2022 October 11>

1 *cytokine/ 66820

2 cytokine.mp. [mp=title, abstract, heading word, drug trade name, original title, device manufacturer, drug manufacturer, device trade name, keyword heading word, floating subheading word, candidate term word] 621984

3 interleukin.mp. 832230

4 *tumor necrosis factor/ 24268

5 *transforming growth factor beta/ 22794

6 adiponectin.mp. [mp=title, abstract, heading word, drug trade name, original title, device manufacturer, drug manufacturer, device trade name, keyword heading word, floating subheading word, candidate term word] 44296

7 1 or 2 or 3 or 4 or 5 1131323

8 epithelial mesenchymal transition/ 57623

9 epithelial mesenchymal transition.mp. [mp=title, abstract, heading word, drug trade name, original title, device manufacturer, drug manufacturer, device trade name, keyword heading word, floating subheading word, candidate term word] 62460

10 *epithelial mesenchymal transition/ 17957

11 "epithelial mesenchymal transformation".mp. 805

12 8 or 9 or 10 or 11 62753

13 *endometrium cancer/ 18233

14 endometrial cancer*.mp. [mp=title, abstract, heading word, drug trade name, original title, device manufacturer, drug manufacturer, device trade name, keyword heading word, floating subheading word, candidate term word] 34204

15 *ovary cancer/ 51456

16 ovarian cancer.mp. [mp=title, abstract, heading word, drug trade name, original title, device manufacturer, drug manufacturer, device trade name, keyword heading word, floating subheading word, candidate term word] 96505

17 *uterine cervix cancer/ 43321

18 cervical cancer.mp. [mp=title, abstract, heading word, drug trade name, original title, device manufacturer, drug manufacturer, device trade name, keyword heading word, floating subheading word, candidate term word] 78869

19 *vulva tumor/ or *vulva cancer/ or *vulva carcinoma/ 6942

20 vulval cancer.mp. [mp=title, abstract, heading word, drug trade name, original title, device manufacturer, drug manufacturer, device trade name, keyword heading word, floating subheading word, candidate term word] 346

21 *vagina cancer/ 1306

22 vaginal cancer.mp. [mp=title, abstract, heading word, drug trade name, original title, device manufacturer, drug manufacturer, device trade name, keyword heading word, floating subheading word, candidate term word] 1233

23 13 or 14 or 15 or 16 or 17 or 18 or 19 or 20 or 21 or 22 224266

24 7 and 12 and 23 276

25 limit 24 to yr="2000 -2022" 276

Ovid MEDLINE(R) ALL <1946 to October 11, 2022>

1 *Cytokines/ 59182

2 cytokine*.mp. [mp=title, book title, abstract, original title, name of substance word, subject heading word, floating sub-heading word, keyword heading word, organism supplementary concept word, protocol supplementary concept word, rare disease supplementary concept word, unique identifier, synonyms] 479600

3 *Interleukins/ 10138

4 interleukin*.mp. [mp=title, book title, abstract, original title, name of substance word, subject heading word, floating sub-heading word, keyword heading word, organism supplementary concept word, protocol supplementary concept word, rare disease supplementary concept word, unique identifier, synonyms] 398900

5 *"Tumor Necrosis Factor Receptor-Associated Peptides and Proteins"/ 292

6 tumour necrosis factor.mp. [mp=title, book title, abstract, original title, name of substance word, subject heading word, floating sub-heading word, keyword heading word, organism supplementary concept word, protocol supplementary concept word, rare disease supplementary concept word, unique identifier, synonyms] 24167

7 *Transforming Growth Factor beta/ 26027

8 TGF.mp. [mp=title, book title, abstract, original title, name of substance word, subject heading word, floating sub-heading word, keyword heading word, organism supplementary concept word, protocol supplementary concept word, rare disease supplementary concept word, unique identifier, synonyms] 85139

9 adiponectin.mp. [mp=title, book title, abstract, original title, name of substance word, subject heading word, floating sub-heading word, keyword heading word, organism supplementary concept word, protocol supplementary concept word, rare disease supplementary concept word, unique identifier, synonyms] 24101

10 1 or 2 or 3 or 4 or 5 or 6 or 7 or 8 773296

11 *Epithelial-Mesenchymal Transition/ 10793

12 "epithelial-mesenchymal transformation".mp. [mp=title, book title, abstract, original title, name of substance word, subject heading word, floating sub-heading word, keyword heading word, organism supplementary concept word, protocol supplementary concept word, rare disease supplementary concept word, unique identifier, synonyms] 620

13 "epithelial-mesenchymal transition".mp. [mp=title, book title, abstract, original title, name of substance word, subject heading word, floating sub-heading word, keyword heading word, organism supplementary concept word, protocol supplementary concept word, rare disease supplementary concept word, unique identifier, synonyms] 34491

14 11 or 12 or 13 34889

15 gynaecological cancer.mp. 1377

16 *Endometrial Neoplasms/ 20004

17 "endometrial cancer*".mp. [mp=title, book title, abstract, original title, name of substance word, subject heading word, floating sub-heading word, keyword heading word, organism supplementary concept word, protocol supplementary concept word, rare disease supplementary concept word, unique identifier, synonyms] 21593

18 *Ovarian Neoplasms/ 74967

19 "ovarian cancer*".mp. [mp=title, book title, abstract, original title, name of substance word, subject heading word, floating sub-heading word, keyword heading word, organism supplementary concept word, protocol supplementary concept word, rare disease supplementary concept word, unique identifier, synonyms] 66311

20 *Uterine Cervical Neoplasms/ 70367

21 "cervical cancer*".mp. [mp=title, book title, abstract, original title, name of substance word, subject heading word, floating sub-heading word, keyword heading word, organism supplementary concept word, protocol supplementary concept word, rare disease supplementary concept word, unique identifier, synonyms] 57642

22 *Vulvar Neoplasms/ 7340

23 "vulval cancers".mp. 33

24 *Vaginal Neoplasms/ 4096

25 "vulval cancer*".mp. [mp=title, book title, abstract, original title, name of substance word, subject heading word, floating sub-heading word, keyword heading word, organism supplementary concept word, protocol supplementary concept word, rare disease supplementary concept word, unique identifier, synonyms] 181

26 15 or 16 or 17 or 18 or 19 or 20 or 21 or 22 or 23 or 24 or 25 223845

27 10 and 14 and 26 206

28 limit 27 to yr="2000 - 2022" 205

CINAHL

Search ID#	Search Terms	Search Options	Actions
S1	(MM "Cytokines")	Expanders - Apply equivalent subjects<br>Search modes - Boolean/Phrase	View Results (8,654)<br>View Details<br>Edit
S2	TX cytokine	Expanders - Apply equivalent subjects<br>Search modes - Boolean/Phrase	View Results (48,899)<br>View Details<br>Edit
S3	(MM "Interleukins")	Expanders - Apply equivalent subjects<br>Search modes - Boolean/Phrase	View Results (8,353)<br>View Details<br>Edit
S4	TX interleukin	Expanders - Apply equivalent subjects<br>Search modes - Boolean/Phrase	View Results (35,575)<br>View Details<br>Edit
S5	TX "tumour necrosis factor"	Expanders - Apply equivalent subjects<br>Search modes - Boolean/Phrase	View Results (2,688)<br>View Details<br>Edit
S6	(MM "Tumor Necrosis Factor")	Expanders - Apply equivalent subjects<br>Search modes - Boolean/Phrase	View Results (6,839)<br>View Details<br>Edit
S7	(MM "Transforming Growth Factor beta")	Expanders - Apply equivalent subjects<br>Search modes - Boolean/Phrase	View Results (961)<br>View Details<br>Edit

S8	TX "Transforming Growth Factor beta"	Expanders - Apply equivalent subjects<br>Search modes - Boolean/Phrase	View Results (3,315)<br>View Details<br>Edit
S9	(MM "Adiponectin")	Expanders - Apply equivalent subjects<br>Search modes - Boolean/Phrase	View Results (1,700)<br>View Details<br>Edit
S10	TX adiponectin	Expanders - Apply equivalent subjects<br>Search modes - Boolean/Phrase	View Results (5,656)<br>View Details<br>Edit
S11	S1 OR S2 OR S3 OR S4 OR S5 OR S6 OR S7 OR S8 OR S9 OR S10	Expanders - Apply equivalent subjects<br>Search modes - Boolean/Phrase	View Results (81,559)<br>View Details<br>Edit
S12	(MM "Epithelial-Mesenchymal Transition")	Expanders - Apply equivalent subjects<br>Search modes - Boolean/Phrase	View Results (258)<br>View Details<br>Edit
S13	TX "epithelial mesenchymal transition"	Expanders - Apply equivalent subjects<br>Search modes - Boolean/Phrase	View Results (2,628)<br>View Details<br>Edit
S14	TX "epithelial mesenchymal transformation"	Expanders - Apply equivalent subjects<br>Search modes - Boolean/Phrase	View Results (50)<br>View Details<br>Edit
S15	S12 OR S13 OR S14	Expanders - Apply equivalent subjects<br>Search modes - Boolean/Phrase	View Results (2,662)<br>View Details<br>Edit
S16	(MM "Endometrial Neoplasms")	Expanders - Apply equivalent subjects<br>Search modes - Boolean/Phrase	View Results (4,365)<br>View Details<br>Edit
S17	TX "endometrial cancer"	Expanders - Apply equivalent subjects<br>Search modes - Boolean/Phrase	View Results (4,589)<br>View Details<br>Edit
S18	(MM "Ovarian Neoplasms")	Expanders - Apply equivalent subjects<br>Search modes - Boolean/Phrase	View Results (11,891)<br>View Details<br>Edit
S19	TX "ovarian cancer"	Expanders - Apply equivalent subjects<br>Search modes - Boolean/Phrase	View Results (12,245)<br>View Details<br>Edit
S20	(MM "Cervix Neoplasms")	Expanders - Apply equivalent subjects	View Results (14,247)

		Search modes - Boolean/Phrase	View Details Edit
S21	TX "cervical cancer"	Expanders - Apply equivalent subjects Search modes - Boolean/Phrase	View Results (15,467) View Details Edit
S22	(MM "Vulvar Neoplasms")	Expanders - Apply equivalent subjects Search modes - Boolean/Phrase	View Results (928) View Details Edit
S23	TX "vulval cancer"	Expanders - Apply equivalent subjects Search modes - Boolean/Phrase	View Results (59) View Details Edit
S24	(MM "Vaginal Neoplasms")	Expanders - Apply equivalent subjects Search modes - Boolean/Phrase	View Results (408) View Details Edit
S25	TX "vaginal cancer"	Expanders - Apply equivalent subjects Search modes - Boolean/Phrase	View Results (145) View Details Edit
S26	S16 OR S17 OR S18 OR S19 OR S20 OR S21 OR S22 OR S23 OR S24 OR S25	Expanders - Apply equivalent subjects Search modes - Boolean/Phrase	View Results (42,531) View Details Edit
S27	S11 AND S14 AND S26	Expanders - Apply equivalent subjects Search modes - Boolean/Phrase	View Results (0) View Details Edit
S28	S11 AND S15 AND S26	Expanders - Apply equivalent subjects Search modes - Boolean/Phrase	View Results (9) View Details Edit

PubMed

(("cytokine*" OR "interleukin*" OR "tumour necrosis factor*" OR "transforming growth factor*" OR adiponectin) AND ("epithelial mesenchymal transformation" OR "epithelial mesenchymal transition")) AND ("gynecological cancer*" OR "endometrial cancer*" OR "ovarian cancer*" OR "cervical cancer*" OR "vulval cancer*" OR "vaginal cancer*") 187

Cochrane (as above) 1

TRIP

epithelial mesenchymal transition/transformation, cytokine, gynaecological cancer 7

Web of Science

cytokines AND (epithelial mesenchymal transition OR transformation) AND (gynaecological cancer OR endometrial cancer OR ovarian cancer OR cervical cancer OR vulval cancer OR vaginal cancer) 11
